# The efficacy and safety of 'antianxiety granule' for anxiety disorder: a multicentre, randomized, double-blind, placebo-controlled, parallel-group trial

**DOI:** 10.1186/s13063-020-4057-1

**Published:** 2020-01-23

**Authors:** Zhongwei Sha, Yiping Hou, Chunchun Xue, Ou Li, Zhimin Li, Huiru Wang, Wenjing Zhang, Jian Xu

**Affiliations:** 1 Department of Mental Diseases, Shanghai Municipal Hospital of Traditional Chinese Medicine, Shanghai University of Traditional Chinese Medicine, Shanghai, 200071 China; 2 Pain Management Centre, Shanghai Municipal Hospital of Traditional Chinese Medicine, Shanghai University of Traditional Chinese Medicine, Shanghai, 200071 China; 3 Standardized Resident Training Staff, Shanghai Municipal Hospital of Traditional Chinese Medicine, Shanghai University of Traditional Chinese Medicine, Shanghai, 200071 China

**Keywords:** Generalized anxiety disorder (GAD), Traditional Chinese medicine (TCM), RCT, Efficacy, Safety

## Abstract

**Background:**

Anxiety disorders are the most prevalent class of lifetime disorders in China, and generalized anxiety disorder (GAD) is one of the most common but frequently overlooked anxiety disorders. Conventional pharmacological treatments for GAD have varying degrees of side effects, dependency, and/or withdrawal syndromes. Traditional Chinese medicine (TCM) is considered a valuable therapeutic option for anxiety disorders and a potentially effective technique to reduce the side effects associated with antipsychotic drugs. This trial aimed to evaluate the clinical efficacy and safety of Antianxiety Granule, a granular Chinese medicine compound, for treatment of GAD.

**Methods/design:**

The current work is a multicentre, randomized, double-blind, placebo-controlled, parallel-group clinical trial with a 6-week treatment schedule. The study consists of three periods: a 1–7-day screening period, a 6-week primary treatment period, and a 1-week follow-up period. Follow-up assessments will be conducted 1 week after the last visit with a face-to-face interview or by telephone. The clinical efficacy of Antianxiety Granule for the treatment of GAD will be evaluated by examining the change in the Hamilton anxiety scale (HAMA) score, state-trait anxiety inventory (STAI) score, and TCM symptom scale in patients with GAD who receive daily TCM treatment. Moreover, an intention-to-treat (ITT) analysis will also be used in this randomized controlled trial (RCT).

**Discussion:**

Our study is a multicentre, randomized, double-blind, placebo-controlled, parallel-group trial to evaluate the safety and efficacy of Antianxiety Granule for the treatment of GAD. The results of this trial will provide valuable clinical evidence for the treatment of GAD.

**Trial registration:**

Chinese Clinical Trial Registry, ChiCTR1800016039. Registered on 8 May 2018.

## Background

Anxiety disorders are the most common lifetime mental disorders. A previous study [1] showed that approximately 3.6% of the global population suffers from an anxiety disorder, and the prevalence of anxiety disorders increased by 14.9% from 2005 to 2015. Because of the comparatively high prevalence, anxiety disorders are a major cause of disability and a substantial disease burden. Nevertheless, anxiety disorders are not taken seriously and are undertreated [2].

Generalized anxiety disorder (GAD) is not well-identified compared with other anxiety disorders [3]. A previous study reported that, even though the combined lifetime prevalence of GAD has been estimated at 3.7%, and it is commonly diagnosed in primary care settings, the lifetime prevalence in China is only 1.0% [4]. Individuals with GAD present with excessive, uncontrollable worry and persistent anxiety that typically lasts for at least 6 months. They continually express fears that they or their families will get sick or suffer an accident, and the feelings are associated with a variety of physical symptoms, such as fatigue, muscular tension, light-headedness, heart palpitations, dizziness, motor restlessness, disturbed sleep, etc. [5, 6]. All of these symptoms can seriously interfere with daily life and may cause functional impairment in patients with GAD. GAD is associated with high rates of comorbid disorders, increasing the risks of mental and physical health conditions [7]. Therefore, increasing social stress and a lack of effective anxiety management will result in greater healthcare resource utilization and an increased economic burden for individuals with GAD.

Currently, the treatment of GAD includes pharmacological and non-pharmacological treatments. Pharmacological treatments include selective serotonin-reuptake inhibitors (SSRIs) and serotonin-norepinephrine reuptake inhibitors (SNRIs), most antidepressants, several benzodiazepines, pregabalin, and buspirone [8]. Antipsychotic medication is the cornerstone treatment for psychotic disorders, but approximately 80% of mental health service users who are taking antipsychotic medication experience numerous, less severe, but nonetheless troubling side effects [9, 10]. Although these drugs exert certain clinical effects, they are associated with varying degrees of side effects, dependence, and withdrawal syndromes; side effects may often vary and be experienced at the same time, including sedation, hypersomnia, insomnia, sexual dysfunction, dry mouth, constipation, urinary problems and dizziness, chronic sedation, and a lack of concentration, all of which interfere with daily life activities [10–12]. Thus, long-term use of these treatments in GAD is discouraged. Patients with GAD may experience reduced benefits and increased harm following the chronic administration of these medications. Patients with anxiety disorders who are concerned about the side effects of pharmacological drugs may preferentially select non-pharmacological treatments, such as applied relaxation, psychotherapy, cognitive therapy, mindfulness interventions, etc. [13–16]. However, it is theoretically difficult to master psychotherapeutic techniques, and mastery of these techniques takes a long time. In China, traditional Chinese herbal medicine (TCHM) has a long history in the treatment of anxiety disorders with certain characteristics. Herbs such as chamomile [17] and *Scutellaria lateriflora* [18] have anxiolytic activities. TCHMs are usually used in combination. Currently, there have been few clinical trials on the efficacy of herbal compounds in individuals with GAD. Because of the treatment effects and few side effects, TCHMs are widely accepted by patients; however, to date there are still not enough standardized, strict, modern, clinical controlled trials on TCM.

In the current study, a multicentre, randomized, double-blind, placebo-controlled, parallel-group clinical trial was designed to evaluate the clinical efficacy and safety of the Chinese medicinal compound Antianxiety Granule for the treatment of GAD.

## Methods/design

### Setting and design

**Fig. 1:**
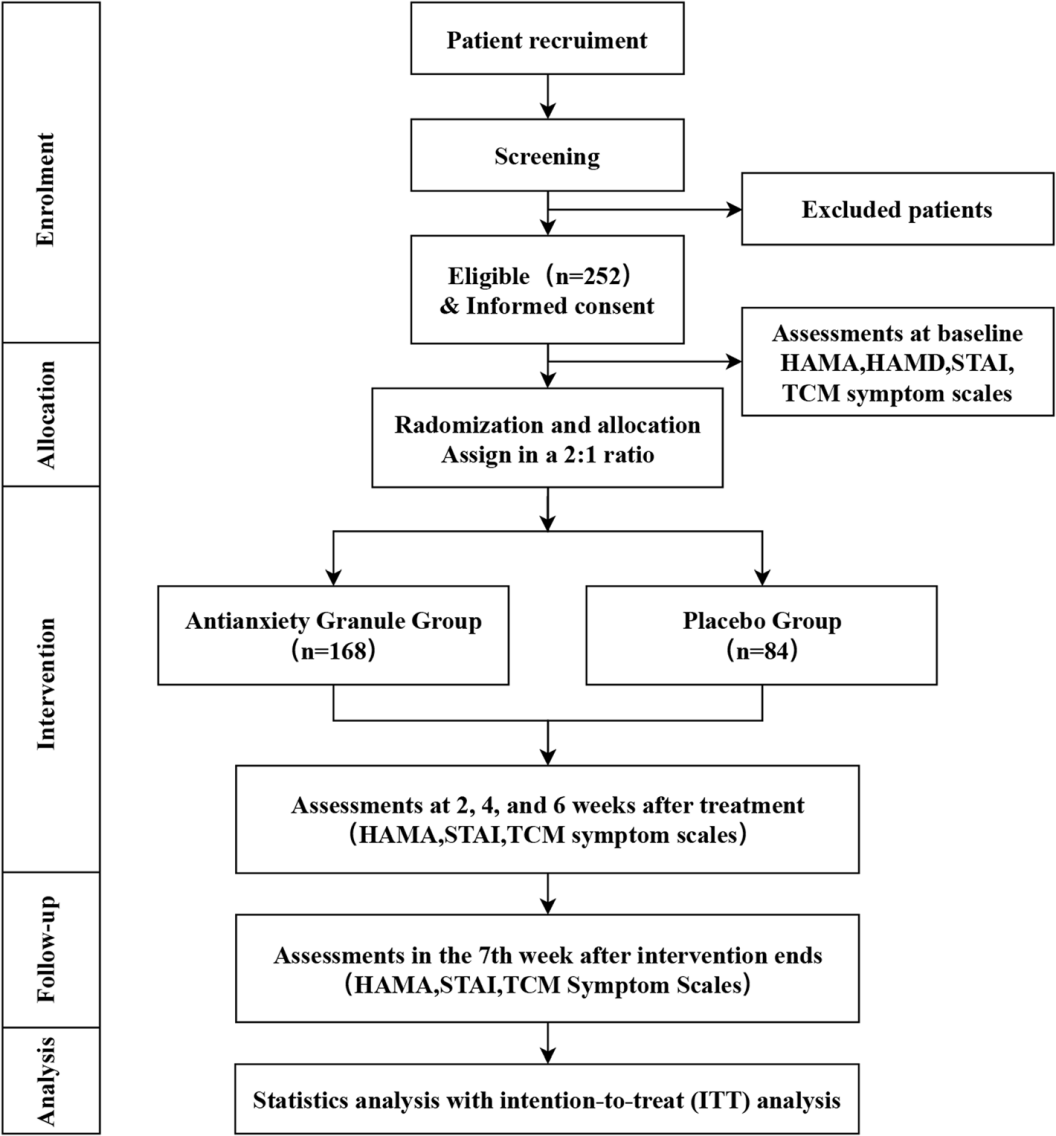
Flowchart of the study trial. HAMA, Hamilton anxiety scale; STAI, State-trait anxiety inventory; TCM, traditional Chinese medicine; ITT, intention to treat

This study is a multicentre, randomized, double-blinded, placebo-controlled, parallel-group, 6-week treatment, clinical trial. Eligible participants will be randomly assigned to an intervention group (Antianxiety Granule) or placebo group at a ratio of 2:1. The participants will be recruited from the following six hospitals: Shanghai Municipal Hospital of Traditional Chinese Medicine, Shanghai Seventh People’s Hospital, Shanghai Chinese and Western Medicine Hospital, The Fifth People’s Hospital of Shanghai, Fudan University, Jingan District Hospital of Traditional Chinese Medicine, and Yangpu District Hospital of Traditional Chinese Medicine. The study consists of three periods: a 1–7-day screening period, a 6-week primary treatment period, and a 1-week follow-up period. Participants will be assessed at baseline and at the 2nd, 4^th^, and 6th week after the initiation of the intervention. Follow-up assessments will be conducted 1 week after the last visit by face-to-face interviews or by telephone (Fig. 1). In this study, the clinical efficacy of Antianxiety Granule will be assessed for the treatment of GAD by examining changes in the Hamilton anxiety scale (HAMA) score, state-trait anxiety inventory (STAI) score and the TCM symptom scale in patients with GAD who receive daily TCM treatment. The flowchart of the study process is presented in Fig. 1, and the timing of the treatment visits and data collection is presented in Table 1.

**Table 1:**
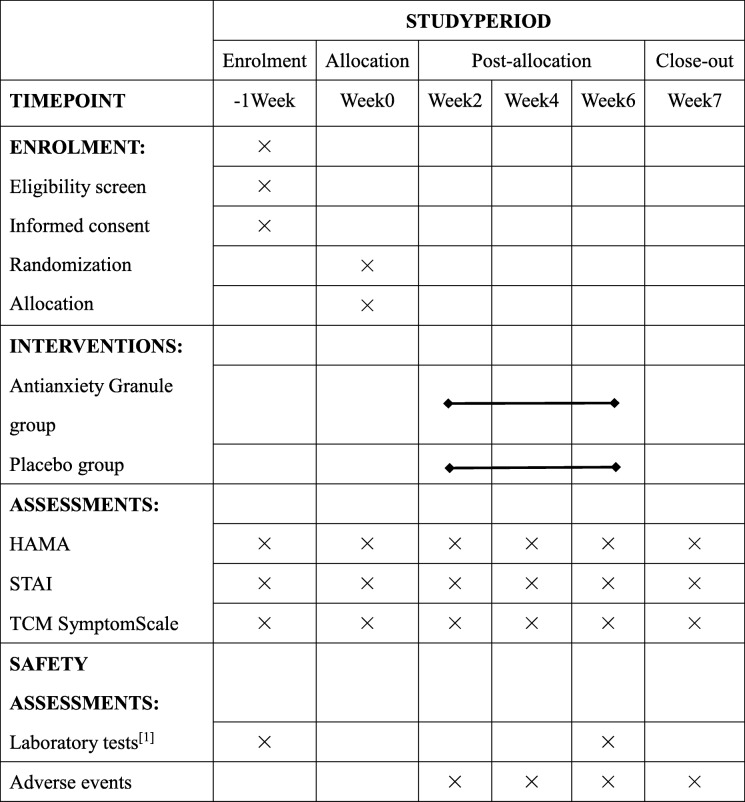
Schedule of enrolment, interventions, and assessments

^a^Laboratory tests: blood, urine, feces, electrocardiogram, kidney and liver function. *HAMA* Hamilton Anxiety Scale, *STAI* State-trait anxiety inventory, *TCM Symptom Scale* traditional Chinese medicine symptom scale

### Participants

#### Inclusion criteria

The study inclusion criteria are as follows:

1. The participant is a male or female outpatient aged 18–70 years
2. The participant meets the diagnosis criteria for GAD (International Classification of Diseases, 10th Revision (ICD–10) and Diagnostic and Statistical Manual of Mental Disorders Fifth Edition (DSM-5th)) and has met the symptom criteria for at least 6 months
3. The participant has never taken anxiolytic agents or has stopped anxiolytic agents for at least half a month
4. The participant has a HAMA score > 14
5. The participant is highly adherent, will take the granule for 6 weeks, and will participate in follow up 1 week later
6. The participant is diagnosed with a TCM syndrome-type according to the *Diagnosis and Treatment Scheme for Anxiety Disorder of TCM*, consistent with the TCM Internal Medicine and the Mental Illness key specialties of the Shanghai Municipal Hospital of Traditional Chinese Medicine
7. The participant participates voluntarily; signed informed consent is required
8. Severity: the participant has experienced poor psychosocial functioning and has feelings of unbearable pain and the inability to escape

#### Exclusion criteria

The exclusion criteria are as follows:

1. The participant has an organic psychosyndrome or an anxiety disorder caused by psychoactive substances, another medical condition (e.g., hyperthyroidism), or non-addictive substances
2. The participant is currently pregnant or lactating or is of reproductive age and is not willing or unable to cease contraceptive use during treatment
3. The participant has obvious heart, liver, kidney, or other systemic symptoms
4. The participant’s laboratory tests and electrocardiogram (ECG) show clinically significant abnormalities, and the investigators shoud judge the results can influence the evaluation of the granule or the participant’s safety
5. The participant’s aspartate aminotransferase (AST) or alanine aminotransferase (ALT) levels exceed two times the upper limit of normal
6. The participant has participated in other drug clinical trials within the previous 4 weeks
7. The participant has a Hamilton depression scale (HAMD) score ≥ 18

### Sample size

A sample size of 252 participants will be recruited on the basis of the pre-post trial. On the basis of previous results and considering an anticipated participant dropout rate of 20%, with a significance level of 0.05, 252 patients will be needed. These 252 patients will be randomly divided into two groups (168 participants in the Antianxiety Granule group and 84 participants in the placebo group, in a ratio of 2:1).

### Recruitment

The participants will be recruited via advertisement posters on bulletin boards in the hospital. If they are willing to participate, they can initially be screened by phone to determine if the basic criteria are met. Then, participants will be further assessed face to face by professional researchers. Once the participants are evaluated with the inclusion or exclusion criteria and volunteer to participate, the researchers will explain the study procedure in detail, and they will be asked to sign a written informed consent form before the intervention begins.

All participants can withdraw their consent at any time during the trial.

### Randomization, allocation, and blinding

The random allocation schedule will be generated by Bingshun Wang, Ph.D., Professor of Medical Statistics, Head, Department of Biostatistics, Shanghai Jiao Tong University School of Medicine (SJTU_SM). Participants will be randomized by computerized, permuted blocks using SAS version 9.4, and stratified by trial centre. The random allocation schedule (including the random numbers and block size) will be sealed in an opaque envelope to conceal group allocation, and allocation concealment will be maintained by the main study administrator/sponsor to avoid selection bias. The randomized allocation schedule and duplicated blinding codes will not be opened during the trial. Simultaneously, a corresponding emergency letter will be prepared for each participant, and the envelope containing the treatment mode will be marked with the participant number. The emergency letter will not be removed unless it is required to do so.

The study is a double-dummy treatment trial. Participants who meet the inclusion criteria will be randomly assigned to an intervention group (Antianxiety Granule) or placebo group in a ratio of 2:1. Neither participants nor attending study personnel will be able to identify the intervention group or placebo group by appearance, packaging, labelling, or shape of the treatment. All the researchers will receive training on the specifications of this study and will be asked to strictly adhere to those specifications.

### Interventions

Eligible participants will receive an Antianxiety Granule or placebo treatment twice daily for 6 weeks according to their random assignment. The Antianxiety Granule is composed of *Cyperi rhizoma*, *Amomum aurantiacum*, *Cinnamomi cortex*, *Curcumae radix*, *Radix salviae*, *Gardeniae fructus*, *licorice*, *Polygala tenuifolia*, *Albizia bark*, *Polygoni multiflori caulis*, and *Daylily.* The herbs will be mixed into brown, bitter, herbal extract granules, and the placebo will consist of one-tenth of the Antianxiety Granule. Both the Antianxiety Granule and placebo will have an identical appearance, colour, and smell. All granules will be packaged in sealed, opaque single-dose sachets with similar labelling. Participants will receive two packaged dose bags (14 doses per bag) every 2 weeks, three times in total, and they will be requested to return the unused granules to the research teams. The use and returning of the granules will be noted on the case report forms (CRFs) by the researchers. All participants will receive some transportation allowances at the end of the trial.

### Outcomes

#### Primary outcome

The primary outcome measure will be a change in the HAMA score [19]. The HAMA scale is the most widely used assessment tool for anxiety disorders in psychiatric clinical and scientific research. The HAMA scale is a 14-item observer-rating questionnaire that can be categorized into two orthogonal groups, “psychic” and “somatic” anxiety. The outcome will be assessed by the difference in the HAMA score from the baseline.

#### Secondary outcomes

Secondary outcomes include the STAI score and TCM symptom scale. The STAI is used extensively in research and provides a reliable self-reported scale for measuring emotional state (S-Anxiety) and personality traits (T-Anxiety) [20]. The STAI consists of two 20-item scales (state anxiety inventory (S-AI) and trait anxiety inventory (T-AI)). The S-AI is used to assess the intensity of anxiety as a transitory emotional state, and the T-AI is used to measure anxiety as a relatively stable personality trait. Each item has a 1–4 score rating. The higher the total score, the more severe the anxiety [21].

The TCM symptom scale will be used to assess the improvement of symptoms but symptom improvement will not be scored. The improvement of symptoms is defined as a reduction in the number of symptoms from enrolment to the end of the study. Each patient is required to record any change in symptoms on the CRF. The researcher will assess the change in symptoms at each visit.

### Safety assessment and adverse events

Safety will be monitored throughout the trial. Routine laboratory safety tests (including routine tests of blood, urine, liver function (ALT, AST), and kidney function (blood urea nitrogen (BUN) and creatinine (Cr)) will be performed at the screening visit and visit 5. Safety will also be assessed through monitoring adverse events (AEs) and vital signs throughout the 6 weeks of treatment. AEs are defined as unexpected symptoms, signs, or diseases that occur during treatment, and all participants will be instructed to report any abnormal event. If AEs happen, the investigators will evaluate the relationship between the AE and the intervention. All AEs will be reported in detail on the CRF. The schedule of enrolment, interventions, assessments, and data collection is presented in Table 1.

### Quality control

The study will be conducted at five hospitals to guarantee its rigour and quality. The investigators will be trained to ensure the quality of the trial. All of the trial data will be fully integrated into the CRFs and monitored by the Clinical Research Centre of Drugs of the Shanghai Municipal Hospital of Traditional Chinese Medicine. Moreover, if any inconsistent data are identified, the primary data will be individually checked by the research monitor and the inconsistency will be reported. The database will be locked after it is reviewed and confirmed by the administrator and the inspector. The source data will be saved appropriately and patient-reported treatment guesses will be obtained to determine the success of blinding. Fisher’s exact test will be used to analyse the data.

### Statistical analyses

The main analysis will follow the intention-to-treat (ITT) principle and will be done as follows: the full analysis set (FAS) will include the randomized participants who received any treatment and completed at least one follow up. The per-protocol set (PPS) will include patients who complied with the protocol. Only when the minimum compliance rate of participants who received the investigational drug is 80% will we use the PPS. Participants who have at least one randomized treatment will be included in the ITT and safety set (SS). To estimate missing primary outcome variables, data from the last study will be used as the final result following the last observation carried forward (LOCF) principle. Data will be analysed using the statistical software SAS version 9.4 or SPSS 20.0.

Sociodemographic variables will be analysed by the *t* test for continuous variables. Matched data will be compared using the Wilcoxon test, and categorical variables will be analysed using the chi-squared test. Change across time of the primary and secondary outcomes will be analysed by repeated measures analysis of variance (ANOVA). We will use ANOVA for randomized block design if the variance and covariance matrix meet the sphericity assumption. ANOVA for repeated measurement data may have two types of error: errors among subjects are the errors corresponding to treatment, and errors within subjects are the errors corresponding to time and the interaction between treatment and time. Significance is will be accepted when the two-sided alpha level is 0.05.

### Clinical trial registration

The trial was registered under the registration number ChiCTR1800016039 (http://www.chictr.org.cn/showproj.aspx?proj=27210) on 8 May 2018. The study was approved by the Ethics Committee of the Shanghai Municipal Hospital of Traditional Chinese Medicine (2017SHL-KY-14).

## Discussion

In the first nationwide survey of mental disorders in China, it was found that anxiety disorders were the most prevalent class of lifetime disorders (7.6%) [22]. GAD is a kind of anxiety disorder that typically begins in adulthood. Onset before puberty is rare, with only 5% of cases developing by the age of 13 years. Onset of GAD before adulthood is uncommon, occurring in fewer than 25% of cases. The clinical course is more persistent in low-income countries. Lifetime comorbidity is high (81.9%), and prevalence estimates vary widely across countries; lifetime prevalence rates in high-income countries are higher than those in middle-income and low-income countries [4]. With rapid economic development in China, Chinese people are more likely to suffer from pressure, anxiety, mood disorders, and related problems. Finding an effective therapeutic method for preventing and combating anxiety disorders is extremely important.

In recent years, several studies have indicated that TCM has a good effect on mental disorders. Antianxiety Granule has been widely prescribed for anxiety symptoms in specialist clinics. The purpose of Antianxiety Granule is to soothe the liver, regulate *qi*, enliven the spleen with aroma, activate the blood and tranquilize the body. In our preliminary studies, Antianxiety Granule, which is composed of *Cyperi rhizoma, Amomum aurantiacum, Cinnamomi cortex, Curcumae radix, Radix salviae, Gardeniae fructus, licorice, Polygala tenuifolia, Albizia bark, Polygoni multiflori caulis and daylily*, has been shown to effectively ameliorate symptoms of anxiety and insomnia in patients with GAD.

In our study, professional researchers are trained to specialize in assessment, which can improve the reliability and generalizability of the results. The trial will be conducted in a clinical outpatient setting with experienced clinicians, and participants will be recruited from the patient base of the other five hospitals involved in the trial. The purpose of this multicentre, randomized, double-blind, placebo-controlled, parallel-group trial is to evaluate the efficacy and safety of Antianxiety Granule compared to a placebo in patients with GAD in Shanghai. The results from the study will provide meaningful information and evidence for clinical practice and to design a confirmative, reasonable RCT in the near future. This study will confirm the safety and efficacy of the TCM Antianxiety Granule and evaluate if Antianxiety Granule can improve distressing symptoms of anxiety.

### Limitations

The RCT still has several design limitations. First, the sample size is relatively small, and the 1-week follow-up phase is short. We will be unable to estimate the recurrence of anxiety after long-term withdrawal. Second, the pathophysiology of GAD has not been clarified; only clinician rating scales, which lack an objective index to judge the effect of TCM treatment on GAD, have been used to measure effects. Finally, the 6-week treatment cycle in this study is relatively short. Bearing these limitations in mind, we will make a more rational treatment cycle and follow-up period to investigate the efficacy of Chinese herbs in patients with anxiety disorder.

## Trial status

This RCT was registered under the number ChiCTR1800016039 (http://www.chictr.org.cn/showproj.aspx?proj=27210) on 8 May 2018. The central ethical approval was obtained from the Ethics Committee of the Shanghai Municipal Hospital of Traditional Chinese Medicine (approval no. 2017SHL-KY-14) on 13 December 2017, and we will not begin recruiting at other centres in the trial until local ethical approval has been obtained. This protocol, version 2, was approved on 2 May 2018. The first participant was randomized in August 2018, and recruitment is ongoing. The final results will be reported in the following year.

## SPIRIT guidelines

Please see Table 1 for a copy of the Standard Protocol Items: Recommendations for Interventional Trials (SPIRIT) figure. The SPIRIT checklist can be found as Additional file 1.

## Supplementary information

**Additional file 1.** SPIRIT 2013 Checklist.

## Data Availability

The datasets used or analysed in the current study are available from the corresponding author upon reasonable request.

## Abbreviations

AEs: Adverse events
ALT: Alanine aminotransferase
ANOVA: Analysis of variance
AST: Aspartate aminotransferase
BUN: Blood urea nitrogen
CRF: Case report form
GAD: Generalized anxiety disorder
HAMA: Hamilton anxiety scale
HAMD: Hamilton depression scale
ITT: Intention to treat
STAI: State-trait anxiety inventory
TCM: Traditional Chinese medicine
